# rhBMP-2 Pre-Treated Human Periodontal Ligament Stem Cell Sheets Regenerate a Mineralized Layer Mimicking Dental Cementum

**DOI:** 10.3390/ijms21113767

**Published:** 2020-05-26

**Authors:** Joo-Young Park, Chan Ho Park, TacGhee Yi, Si-na Kim, Takanori Iwata, Jeong-Ho Yun

**Affiliations:** 1 Department of Oral and Maxillofacial Surgery, Seoul National University Dental Hospital, Seoul 03080, Korea; bbyoung1@snu.ac.kr; 2 Department of Dental Biomaterials, School of Dentistry and Institute for Biomaterials Research and Development, Kyungpook National University, Daegu 41940, Korea; chanho@knu.ac.kr; 3 SunCreate Co., Ltd., Uijeongbu, Gyeonggi-do 11813, Korea; tgwise@naver.com; 4 Drug Development Program, Department of Biomedical Sciences, Inha University School of Medicine, Incheon 22332, Korea; tlsk1206@naver.com; 5 SCM Lifescience Co., Ltd., Incheon 22332, Korea; 6 Department of Periodontology, Graduate School of Medical and Dental Sciences, Tokyo Medical and Dental University, Tokyo 113-8549, Japan; iwata.peri@tmd.ac.jp; 7 Department of Periodontology, College of Dentistry and Institute of Oral Bioscience, Jeonbuk National University, Jeonju 54896, Korea; 8 Research Institute of Clinical Medicine of Jeonbuk National University-Biomedical Research Institute of Jeonbuk National University Hospital, Jeonju 54907, Korea

**Keywords:** dental stem cells, stem cell therapy, mesenchymal stem cell, regeneration, tissue engineering

## Abstract

The periodontal complex consisting of alveolar bone, cementum, and periodontal ligaments (PDL) supports human teeth through the systematic orchestration of mineralized tissues and fibrous tissues. Importantly, cementum, the outermost mineralized layer of dental roots, plays an essential role by bridging the inner ligaments from the dental root to the alveolar bone. When the periodontal complex is damaged, the regeneration of each component of the periodontal complex is necessary; however, it is still challenging to achieve complete functional regeneration. In this study, we tried to control the regeneration of cementum and PDL by using a human PDL stem cell (hPDLSC) sheet engineering technology with the pretreatment of recombinant human BMP-2 (rhBMP-2). Isolated hPDLSCs obtained from extracted human teeth were pretreated with rhBMP-2 for in vitro osteogenic differentiation and grafted on the micro/macro-porous biphasic calcium phosphate (MBCP) blocks, which represent dental roots. The MBCPs with hPDLSC sheets were implanted in the subcutaneous layer of immune-compromised mice, and rhBMP-2 pretreated hPDLSC sheets showed higher mineralization and collagen ligament deposition than the no-pretreatment group. Therefore, the rhBMP-2-hPDLSC sheet technique could be an effective strategy for the synchronized regeneration of two different tissues: mineralized tissue and fibrous tissues in periodontal complexes.

## 1. Introduction

Human teeth are embedded inside the jawbone, specifically inside the subarea of the jaw called alveolar bone. Alveolar bone holds each tooth according to its morphologic shape [1] but also does using functional ligaments directly connecting the tooth and bone (periodontal ligaments, PDL) [2]. As the tooth roots need an apparatus to tightly hold the PDL, they have cementum, the outmost root layer where PDLs are anchored [3]. The anchored collagenous tissue fibers (Sharpey’s fibers) in cementum sprout toward adjacent alveolar bone in several different directions [4]. In this way, the periodontal complex, which consists of cementum, PDL, and alveolar bone, provides significant support to tooth structures and prevents tooth damage in occlusal or masticatory loading environments [5].

When the periodontal complex is infected by pathogenic oral bacteria, each element of the periodontal complex is damaged, which eventually results in the disease status called periodontitis. The clinical symptoms of periodontitis are gum bleeding, bad oral odor, alveolar bone destruction, and inflammation of periodontal ligaments, which results in tooth mobility and loss of teeth. Although chronic periodontitis is prevalent in adults and seniors worldwide, a definitive method to regenerate each element of the periodontal complex has not been identified, especially how to regenerate cementum, including the embedded functional PDLs.

The reason why it is difficult to regenerate cementum with PDL is due to the complexity of the tissues themselves [4]. The periodontal complex is a combination of two different hard tissues—alveolar bone and cementum—and with soft tissue ligaments attaching inside those hard tissues: the periodontal ligaments. As a result of the spatiotemporal manner of the tissue development, it is difficult to achieve complete physiological regeneration of the periodontal complex, and various approaches have been previously developed. For example, gene therapy or biologic delivery [6,7], stem cell differentiation [8,9], and biomaterial-based cementogenic control [3,10,11] have been tried to mimic the developmental processes of periodontal tissue formation. The key process during regeneration is formation of the cementum layer with embedded PDL connecting into the alveolar bone. However, there has been no definitive method that completely regenerates embedded fibers in the outmost layer of a dental tooth, i.e., cementum.

Among those trials, the cell sheet technique was demonstrated in 2004 by the Yamato and Okano group based on the necessity of synthetic material free approaches for tissue regeneration [12]. The cell sheet technique is based on a temperature-responsive polymer known as poly(N-isopropylacrylamide) (PIPAAm). When PIPAAm is grafted onto the surface of a cell culture dish, temperature changes can reversibly determine hydrophilic or hydrophobic characteristics of the polymer that produce mono-layered cell sheets [12]. A simple temperature change can allow cell sheet harvest with intact cell-to-cell junctions, while a general cell-harvesting method used trypsin for the enzymatic degradation of extracellular matrices. Using this technology, the regeneration of diseased or damaged tissues has been tried. Various cell types can be cultivated on PIPAAm-treated culture dishes in vitro, and cell sheet constructs can be transplanted to induce target tissue regeneration [13,14]. Since the cell sheet technique provides a cell-rich environment with a possible three-dimensional structure without using scaffold materials, pre-clinical and clinical achievements have been reported for various tissue regeneration needs, such as in the urinary tract, myocardia, and cornea [15,16]. 

The most frequently used cellular resources for the cell sheet technique are mesenchymal stem cells (MSCs). MSCs are adult stem cells that can differentiate into multiple lineages of tissues. Therefore, they are well known to show a therapeutic potential for tissue regeneration, such as the regeneration of bone, cartilage, fat, and fibrous tissues [17]. Among the MSCs, those obtained from the extracted teeth are dental MSC,s and they can be isolated ethically and non-invasively [18]. Dental pulp stem cells, PDL stem cells (PDLSCs), dental follicle progenitor cells (DFPCs), and most recently discovered periapical cyst-MSCs (PCy-MSCs) are dental MSCs, and they showed extensive proliferative potential and the ability to differentiate into various cell types such as osteoblasts, adipocytes, and neurons [17,18,19]. Using the dental MSCs, the application of the cell sheet technique for dental tissue regeneration was also tried, and Iwata et al. used three-layered cell sheets to regenerate alveolar bone and PDL structures around periodontal defects in pre-clinical and clinical scenarios [15,16,20]. Although this technique enabled alveolar bone regeneration and PDL formation around tooth root surfaces, collagen formation in cementum with its morphological integrations for periodontal complexes were difficult to achieve. 

To break through those difficulties, we investigated a new strategy for cementum regeneration using recombinant human bone morphogenetic protein-2 (rhBMP-2) pre-treated hPDLSC sheets. With micro/macro-porous biphasic calcium phosphate (MBCP) blocks, which mimic dental roots, grafted rhBMP-2 pre-treated hPDL stem cells (hPDLSCs) could promote the mineralized layer and enable PDL formation simultaneously. In our previous study, BMP-2 could promote mineralized tissue formation upon the transduction of individual hPDLSCs, which have the potential for multipotent differentiation [6]. Although BMP-2 treatment is a promising strategy for the osteogenic differentiation of hPDLSCs, the predictable, controlled differentiation of individual cells was hard to achieve. Therefore, in this study, we investigated if rhBMP-2 pre-treated hPDLSC sheets could effectively promote the regeneration of the mineralized layer with embedded PDL to promote functional human dental cementum and the periodontal complex. 

## 2. Results

### 2.1. The hPDLSC Clones Express MSC Markers and Show Multipotent Differentiation and Immunosuppression Abilities

**Figure 1:**
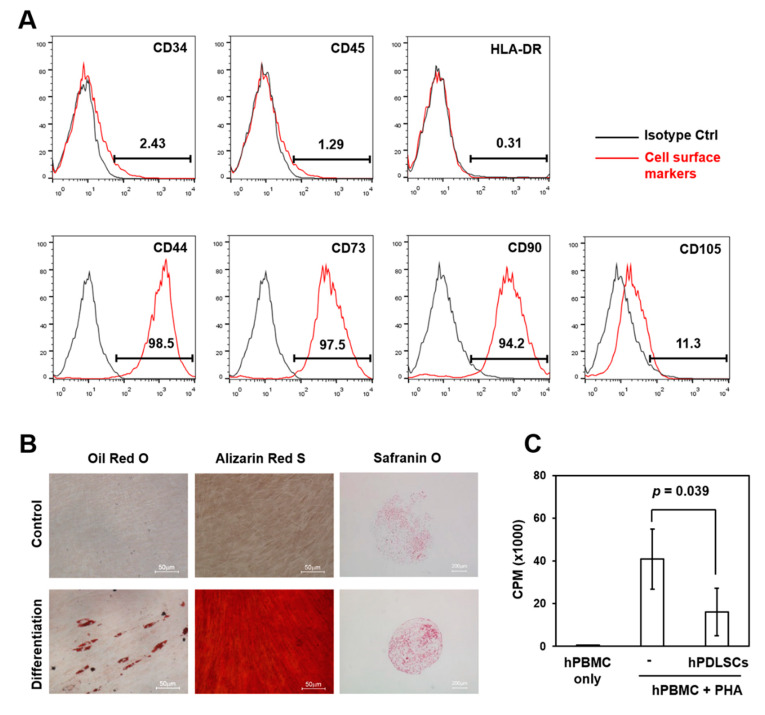
Characterization of human periodontal ligament stem cells (hPDLSCs). (**A**) hPDLSCs were analyzed by flow cytometry with mesenchymal stem cell (MSC) markers. The numbers showed the frequencies of positive staining of the antibodies for cell surface markers (red lines) compared to the isotype controls (black lines). (**B**) The multilineage potentials for MSC differentiation were evaluated in vitro based on adipogenic, osteogenic, and chondrogenic differentiation, visualized by oil red O, alizarin red S, and safranin O stainings, respectively. (**C**) The in vitro immunosuppression of human lymphocyte proliferation by hPDLSCs was evaluated with mitogen-activated human peripheral blood mononuclear cells (hPBMCs). The hPBMCs were stimulated with phytohemagglutinin (PHA; 1 μg/mL) and co-cultured with hPDLSCs (cell number ratio of hPDLSCs: PBMCs = 1:5) for three days. Lymphocyte proliferation was determined based on [3H]-thymidine incorporation, and data are shown with their statistical significance (mean ± SD; * *p* = 0.039).

A single colony of hPDLSCs was obtained in a subfractionation culture, and a few clones were randomly selected. The selected clones were characterized for cell surface marker expression, differentiation potential, and immunosuppression ability based on the criteria for MSCs [21]. The hPDLSC clones expressed MSC-associated markers CD44, CD73, CD90, and CD105 (Figure 1A), but they did not express hematopoietic stem and hematopoietic cell markers CD34, CD45, and HLA-DR (Figure 1A). Moreover, the hPDLSC clones were differentiated into adipose, bone, and chondrogenic tissues under each differentiation condition, indicating that they possess multilineage differentiation potentials (Figure 1B). The immunoregulatory property is another characteristic of MSCs [22,23], and the hPDLSC clones indeed inhibited lymphocyte proliferation shown in the in vitro immunosuppression assay (Figure 1C). These data show that the hPDLSC clones in this study were MSCs that were suitable for the development of cell sheets. 

### 2.2. In Vitro Study for Mineralization of hPDLSC Sheets after rhBMP-2 Pre-Treatments

**Figure 2:**
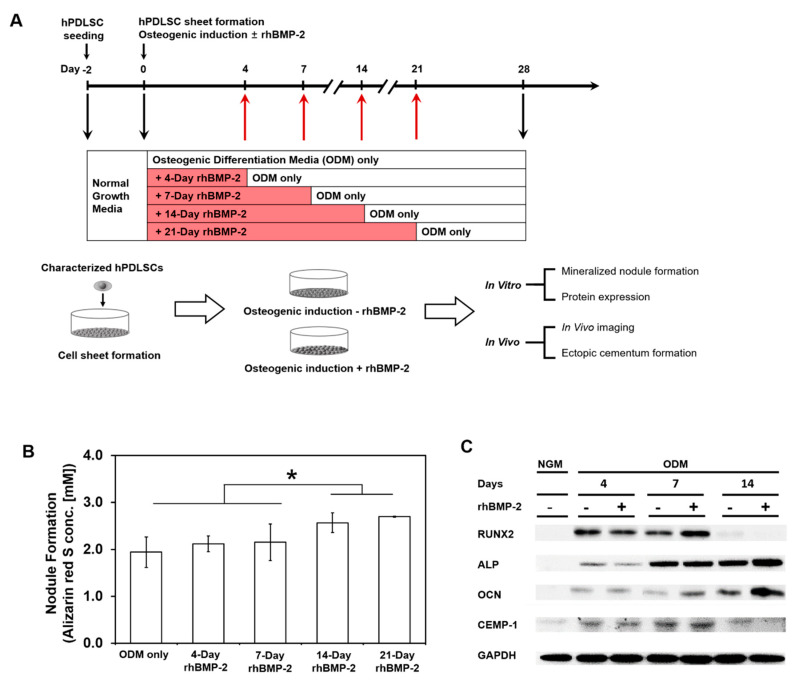
Experimental design scheme and quantification of mineralized nodule formation from hPDLSC sheets after a time series of recombinant human BMP-2 (rhBMP-2) treatments. (**A**) The timeline displays the various time points of rhBMP-2 treatment for the in vitro and in vivo experiments. (**B**) Osteogenic differentiation was quantified based on alizarin red S staining for the mineralized nodules, and the optimal time point of rhBMP-2 treatment was determined through the cultivation of hPDLSC sheets in osteogenic differentiation medium (ODM) for up to 28 days. Mineral nodule formation was semi-quantified through alizarin red S staining. (**C**) The enhancement of osteogenic potential was evaluated through Western blot analysis of runt-related transcription factor 2 (RUNX2), alkaline phosphatase (ALP), osteocalcin (OCN), and cementum protein-1 (CEMP-1) expression. Glyceraldehyde 3-phosphate dehydrogenase (GAPDH) was used as a loading control.

The optimal osteogenic conditions for hPDLSC sheets were investigated with/without 100 ng/mL rhBMP-2 pre-treatment in osteogenic differentiation medium (ODM) for the first 4, 7, 14, or 21 days, followed by ODM alone until 28 days (Figure 2A). Alizarin red S staining was used to evaluate mineral nodule formation. Osteocalcin (OCN), alkaline phosphatase (ALP), runt-related transcription factor 2 (RUNX2), and cementum protein-1 (CEMP-1) were used as markers of osteogenic and cementogenic protein expression. When the degree of mineralization was quantified with the dissolved nodules, the amount of mineralization at 21-day treatment show no significant difference compared to the amount at 14-day treatment. However, these groups differed significantly from the other three groups: ODM only, 4-day and 7-day treatments (Figure 2B). Therefore, 14-day rhBMP-2 pretreatment was applied for the later in vivo experiments. In terms of osteogenic and cementogenic potential, protein levels of RUNX2 and CEMP-1 were higher when hPDLSC sheets were pretreated with rhBMP-2 for 7 days, while the amounts of ALP and OCN proteins were higher at 14-day treatment (Figure 2C). 

### 2.3. In Vivo Study for Mineralized Layer Deposition on MBCP Blocks with Collagenous Fiber Formation 

**Figure 3:**
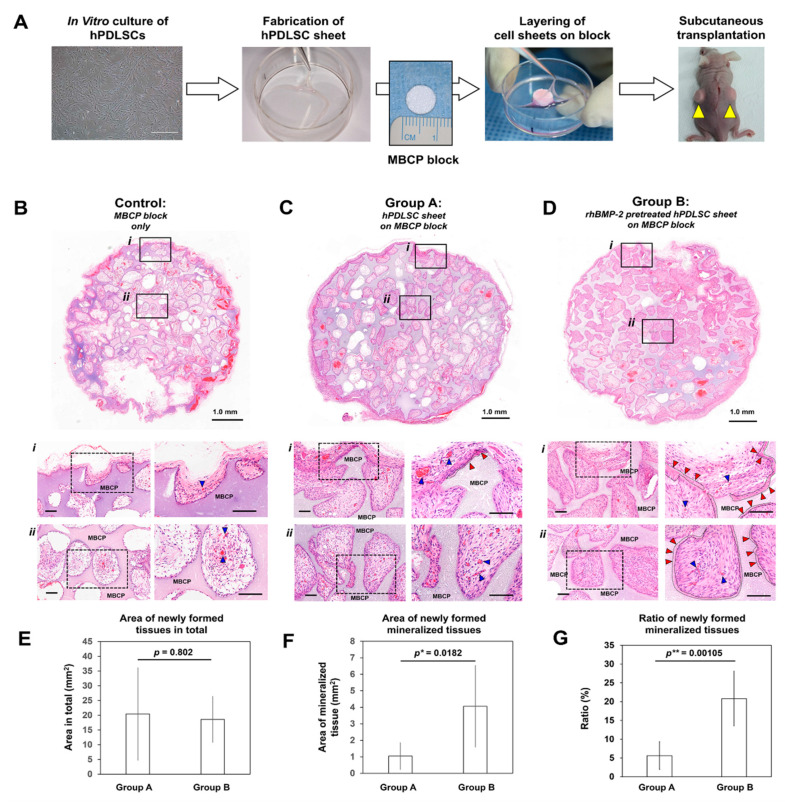
Transplantation of rhBMP-2 pretreated hPDLSCs on micro/macro-porous biphasic calcium phosphate (MBCP) blocks in immunocompromised mice and histomorphometric analysis of mineralized tissue regeneration. (**A**) Flow chart demonstrating the transplantation procedure in an immunocompromised mouse model. After hPDLSCs were cultivated and hPDLSC sheets were fabricated, the three-layered sheets were combined with MBCP blocks, and the complexes (hPDLSC sheet on MBCP block and rhBMP-2-hPDLSC sheet on MBCP block) were subcutaneously transplanted. (**B**–**D**) The three different groups were harvested at the 4-week time point, and hematoxylin and eosin (H&E) staining was performed for qualitative and quantitative assessments. Compared with the MCBP block-only group, the two groups with hPDLSC sheets displayed mineralized tissue formation around the blocks (red arrows) and effective tissue infiltration with angiogenesis (blue arrows). In particular, the rhBMP-2 pretreated hPDLSC sheets highly promoted mineralized layer deposition on the MCBP block surface with critical thickness and facilitated fibrous tissue formation with tissue integration (red arrows). Scale bars at the top figures represent 1.0mm. Scale bars at the middle and the bottom represent 100 μm. (**E**–**G**) The area of newly formed tissues in total was measured as the total tissue area subtracted by the MBCP block area. The area of mineralized tissue was identified as shown in black dotted lines by two different authors, and the absolute areas were measured. The difference between groups A and B was evaluated for the absolute area and the ratio of mineralized tissue in total newly formed tissues. The group B, rhBMP-2 pretreated hPDLSC sheet showed significantly increased amount of mineralized tissue on the outer and inner surface of MBCP blocks, although the newly formed tissue areas in total had no difference between the groups. Statistical significance was shown as * *p* < 0.05 and ** *p* < 0.005.

Histomorphometry was performed on the MBCP block surface layer adjacent to the hPDLSC sheets (Figure 3B–D: area i) and inside the blocks (Figure 3B–D: area ii) for the following three groups: (1) Control: MBCP block only, (2) Group A: hPDLSC sheet on MBCP block, and (3) Group B: rhBMP-2 pretreated hPDLSC on MBCP block. Biocompatible MBCP blocks in the control group formed blood vessels, but they exhibited no mineral deposition on MBCP surface (Figure 3B, blue arrows). In Groups A and B (Figure 3C,D), mineralized tissue formation on the outer and inner surfaces the MBCP (red arrows) was both qualitatively and quantitatively validated through hematoxylin and eosin (H&E) staining (Figure 3C–G). Histomorphometrical analysis showed that the amounts of newly formed tissue in total had no difference between groups A and B (Figure 3E), but the absolute area and percentage of mineralized tissue formation differed significantly between Group A and B (Figure 3F,G). 

**Figure 4:**
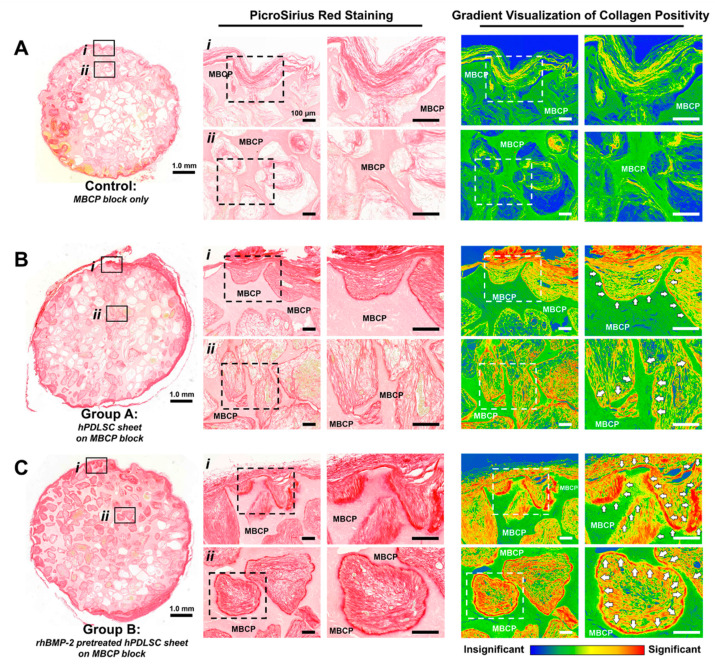
Analyses of collagen formation in rhBMP-2 pretreated hPDLSCs transplantation model. (**A**–**C**) The outer layer (i) and inner structure (ii) of MBCP blocks were stained by PicroSirius red and qualitatively analyzed for collagen deposition at different magnifications (middle column). Density gradients of PicrosSirius staining was further analyzed, and the amount of collagen deposition on the MBCP blocks was evaluated. (**A**) The MBCP blocks alone (control) displayed limited collagen formation around the surface, while the rhBMP-2 pre-treated hPDLSC sheet group (group **C**) exhibited intense PicroSirius red staining, indicating that collagen fibers were newly generated with high density and in significant thickness inside the area of mineralized layer in contrast to the group **A** and **B**. In particular, semi-quantification analysis based on color profiles from the density gradients demonstrated that rhBMP-2 pretreated hPDLSC sheet group showed collagen matrix formation inside the MBCP block as well as the outside of the block, and the collagen fibrous tissues were aligned perpendicularly or obliquely to the mineralized layers (white arrows, red color represents significantly high). Scale bars at the very left column represent 1.0mm. Scale bars at the middle and the right columns represent 100 μm.

For the determination of collagen formation, PicroSirius red staining was utilized for the morphological colorimetric visualization of collagen positivity in Controls, Group A, and Group B (Figure 4). Collagenous fiber structures were identified on the outer surface of MBCP blocks and thick collagen depositions with strong intensity on the pore surface inside MBCP blocks were assessed quantitatively (Figure 4B,C). After rhBMP-2 pre-treatment, hPDLSC sheets promoted significant collagen deposition for mineralized layer formation, as evidenced by the thick red regions in the density gradient profiles (Figure 4C). Based on tissue integrations with oriented fibrous tissues as well as collagenous architecture and mineralized layers by infiltrated tissues, rhBMP-2 pretreatment of hPDLSC sheets could promote significant PDL-cementum-like complex formation in periodontal-mimetic microenvironments (Figure 4). 

**Figure 5:**
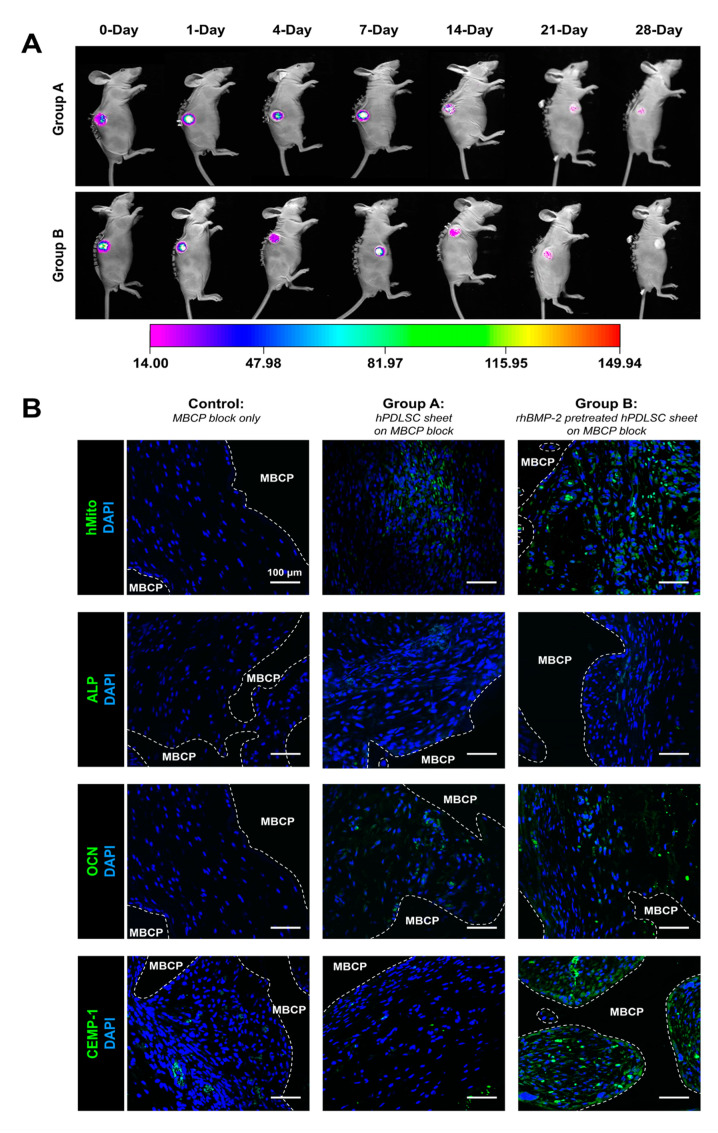
Viability profiles obtained through in vivo bioluminescence imaging and immunohistochemistry of the hPDLSC sheets in the transplanted groups. (**A**) The hPDLSC sheets were cultured and fabricated in two different conditions; group A in osteogenic differentiation medium (ODM) or group B in ODM with rhBMP-2 and loaded on MBCP block, then transplanted in the immunocompromised mice models. Bioluminescence imaging was performed for the transplantation in a time series and qualitatively demonstrated that hPDLSC sheets had long-term viability in physiological environments. (**B**) All the groups displayed positive nuclear staining with 4′,6-diamidino-2-phenylindole (DAPI), demonstrating that the host tissues invaded the MBCP blocks. The viability of the transplanted hPDLSC sheets was assessed based on human mitochondrial ribosomal protein L11 (hMito) expression, which was significant in groups A and B (hPDLSC sheet on MBCP and rhBMP-2 pretreated hPDLSC on MBCP blocks). For early and late osteogenic differentiation, ALP and OCN levels were analyzed. Although ALP expression (marking the early stage of osteogenesis) was similar among the three groups, OCN expression (marking the late stage of osteogenic differentiation) at 4 weeks was different in groups A and B than in the control. CEMP-1 staining was much stronger in group B (rhBMP-2 pre-treatment) than in the other groups, demonstrating that rhBMP-2 treatment can promote significant cementum-like tissue formation and mineral deposition on the MBCP block surface. Scale bars represent 100 μm.

The viability and sustainability of transplanted hPDLSC sheets were evaluated for transplantation efficacy. To detect the viability of hPLDSC sheets after transplantation in vivo, bioluminescence by the luciferase-expressing hPDLSC sheets was monitored according to the post-transplantation days (Figure 5A). Bioluminescence signals were detected from the day 0 until the post-transplantation day 28 in both groups A and B, implying the viability of the transplanted hPDLSCs for 4 weeks, although the signals became gradually diminished (Figure 5A). To assess the efficacy of transplantation, antibody for human mitochondrial ribosomal protein L11 (hMito) was stained for tissue samples at 4 weeks of transplantation. hMito positive cells were supposed to be human originated hPDLSCs that were distinct from the host murine cells, and the tissues from the groups A and B exhibited hMito positivity while the control group showed negative staining (Figure 5B). To further investigate the regeneration outcome of the transplanted hPDLSCs, expressions of cementogenic and osteogenic markers were evaluated. The expression of CEMP-1, which indicates the activity of cementoblasts and their progenitors [6,24] in group B, demonstrated that rhBMP-2-hPDLSC sheets promoted cementum-like mineralized structure formation (Figure 5B). In terms of osteogenic differentiation, ALP expression for the early stage was not detectable in any groups, but OCN expression for the late stage differed between the control group and the hPDLSC sheet groups (Figure 5B). 

## 3. Discussion

Various tissue-engineering strategies have been developed to promote bone–PDL complex formation in pre-clinical or clinical scenarios [2,4,5,10,25,26,27,28,29]. While different approaches have enabled the neogenesis of multiple periodontal tissues with spatial compartmentalization and structural similarity to natural complexes, it has still been challenging to reconstruct functional periodontal complex [30]. Cell sheet engineering is one promising technique to achieve biological integration and spatiotemporal tissue-specific classification for periodontal tissue engineering [2,13,15,31]. One of the reasons for successful periodontal regeneration by cell sheet engineering is the high flexibility of the sheet itself. Multi-layered PDLSC sheet constructs could spatially access surgically created periodontal defects and structurally adapt to treated tooth–root surfaces because of its flexibility [15]. The flexibility is critical for periodontal tissue regeneration because the geometry of periodontal defects is highly unpredictable. Besides, each individual tooth has its own anatomical variance, which needs customized adaptation of the grafted materials [28]. In addition, three-layered constructs with approximately 60 μm thickness (single hPDLSC sheet thickness: 20 μm) were reported to spatially secure a 100-μm PDL interface without ankylosis (bone fusion to the tooth–root surface) [27,28]. Based on these properties, which are comparable with biomaterial-based scaffolding systems, biomaterial-free PDLSC sheets could provide high accessibility to various geometric defect sites for periodontal tissue regeneration.

For the functioning restoration of periodontal complexes with physical responses against mastication or occlusion, cementum is crucial for anchoring fibrous connective PDLs onto the tooth–root surface with Sharpey’s fibers, which are the terminal ends of collagenous PDL fibers [32]. Therefore, the cementogenic differentiation of dental stem cells and the deposition of minerals on the tooth–dentin surface are key for functional tissue regeneration [3]. In this study, hPDLSC sheets were pretreated with rhBMP-2 for 14 days, and three-layered cell constructs were prepared with optimal viability [13,15,33]. The mineralized layer was deposited on MBCP blocks with a compositional similarity to dentin microenvironments, even though the MBCP had architectural differences from natural tooth dentin [34]. In addition to the compositional similarity, because MBCP blocks have less individual variation than natural teeth from different human subjects, they can be used as a dentin substitute. Interestingly, after assembling with MBCP blocks, rhBMP-2 pretreated hPDLSC sheets formed cementum-like mineralized tissue, and collagen fibers inserted perpendicularly or obliquely to the MBCP surface. 

Therefore, rhBMP-2 pre-treatment promoted the collagen fiber formation from the hPDLSC sheets and cementogenic differentiation concomitantly. These results are encouraging because the addition of certain growth factors failed to induce the harmonized regeneration of two different tissues, which was mainly because of difficulty in release control. Growth factors have been previously studied for sustained release [35]; however, various complications have been reported including different degrees of ankylosis around the tooth–root surface (BMPs), early stages of osteoblastic stimulation (transforming growth factor-β [TGF-β] or platelet-derived growth factor [PDGF]), and toxicity at required pharmacological dosages [10,36]. Therefore, practical control and modulation of the growth factors are required to optimally regulate the PDL regeneration with limited cementogenic differentiation for spatial compartmentalization of periodontal complexes. 

Based on our previous study [6], rhBMP-2 pretreatment can physiologically activate hPDLSC sheets with osteogenic potential. In this study, collagen networks in deposited mineralized layers on the MBCP block surface were identified by PicroSirius red staining and the layer thickness was semi-qualitatively analyzed through gradient visualization of collagen positivity. Moreover, fibrous tissues were inserted into the newly formed mineralized layers with oblique/perpendicular angulations, and fiber–mineral tissue complexes developed morphological tissue integrations. Therefore, although the mouse subcutaneous model system possessed limited bioactive components of periodontal tissues, rhBMP-2 pretreatment effectively promoted periodontal-like interfacial tissue complex neogenesis between fibrous tissues and cementum-like mineral deposition on the MBCP block surface. Additionally, the MBCP block was used as a dentin substitute in this study and did not release rhBMP-2 by themselves. Therefore, the differences in collagen fiber generation and mineralization on the MBCP block between group A and group B (Figure 3 and Figure 4) should result from the rhBMP-2 pretreatment on hPDLSC sheets. New technologies of drug carrier materials are available such as rhBMP-2 delivering nanomaterials for bone regeneration [37]; however, they still need to be developed sophisticatedly for regeneration of the periodontal complex.

Our previous study also demonstrated that the rhBMP-2 treatment of individual hPDLSCs (not hPDLSC sheets) had significant osteogenic differentiation potential [6]. Iwata et al. previously reported that ODM pretreatment of hPDLSC sheets for 14 days significantly induced osteogenesis [33]. However, in this study, rhBMP-2-hPDLSC sheets expressed relatively low levels of ALP and OCN to characterize osteogenic differentiation. The weak expression of ALP at 4 weeks was reasonable, because ALP could be expressed in the early stage of mineralization. Interestingly, OCN expression was relatively low in all the groups in this study, even though OCN is expressed in the late phase of the bone formation process. Nonetheless, based on the significantly higher expression of CEMP-1 in group B, it was remarkably evident that the rhBMP-2 pretreated hPDLSC sheets enhanced cementogenic differentiation and promoted cementum-like tissue formation on MCBP blocks in immunohistochemical analysis. Thus, rhBMP-2 pretreatment prior to transplantation could activate cementogenic differentiation in hPDLSC sheets for mineralized layer deposition on MCBP blocks rather than osteogenic differentiation. 

This study demonstrated that rhBMP-2 pretreatment qualitatively and quantitatively improved the fate of hPDLSC sheets by promoting both cementum-like and fibrous tissue formation in vitro and in vivo. Although the subcutaneous transplantation mice model still has limitations in controlling calcified tissue formation and in ligament orientations, our rhBMP-2-hPDLSC sheets exhibited promising performance in forming mineralized layers with fiber insertions on MBCP blocks with compositional similarity to tooth dentin. For the clinical application of the rhBMP-2 pretreated hPDLSC cell sheet technique for human periodontal regeneration in the future, the cultivation of hPDLSCs ex vivo should be performed without xenogenic serum. Therefore, a supplement nutrient such as human autologous platelet lysate can be applied, or a serum-free cultivation method should be developed for preventing the host immune responses [19,38,39].

In conclusion, our developed strategy can promote physiological periodontal tissue formation on dentin-exposed teeth in vivo and integrate fibrous connective tissues in specific orientations for functional restoration under mastication or occlusion.

## 4. Materials and Methods

### 4.1. Isolation of Single Colony hPDLSCs for Cell Sheet Development and Phenotyping of Adult Stem Cells

For the isolation and culture of primary hPDLSCs, PDL tissues from extracted premolars were harvested from healthy patients, following a protocol approved by the Institutional Review Board of Jeonbuk National University Hospital (CUH 2015-06-038-001). A modified subfractionation culture method [40,41] was used to isolate stem cells to produce highly homogeneous clonal stem cells. Briefly, the root surface of the extracted tooth was scraped to collect PDLs, which were then digested with 2 mg/mL collagenase (Wako Pure Chemical Industries, Tokyo, Japan) and 1 mg/mL dispase (Gibco BRL, Grand Island, NY, USA). Single colony-forming-unit-derived clones were separated and cultured as previously described [8,40,41]. Then, hPDLSCs were cultured in normal growth medium: alpha minimum essential medium (α-MEM, Gibco), 10% fetal bovine serum (FBS, Gibco), 100 mM ascorbic-acid-2-phosphate (Sigma-Aldrich), 100 U/mL penicillin, and 100 mg/mL streptomycin (Gibco BRL). Cultured hPDLSCs from passage five to eight were used for this study. 

For the characterization of adult stem cells, hPDLSCs were incubated with fluorochrome-conjugated antibodies for the cell surface proteins CD45, CD90, CD105, HLA-DR, CD44, CD73 (all from BD Biosciences, San Diego, CA, USA), and CD34 (AbD Serotec, Oxford, UK). Flow cytometry data were acquired with FACSCalibur (BD Biosciences, San Jose, CA, USA) and analyzed by FlowJo software (FlowJo, USA) to detect the positive staining of antibodies compared to the isotype-matched negative control antibodies, as described previously (Kim et al., 2016). With the characterized hPDLSCs, cell sheets were developed on thermoresponsive culture dishes (Nunc™ Dishes with UpCell™ Surface, ThermoFisher Scientific, Rochester, NY, USA), as previously described [20,33].

### 4.2. Analysis of Differentiation and Immune Suppression Abilities of hPDLSCs

The hPDLSCs were cultured in three different conditions—adipogenic, osteogenic, or chondrogenic differentiation media—in accordance with a previous study [42]. Oil red O, alizarin red S, or safranin O were stained to assess adipogenesis, osteogenesis, or chondrogenesis of hPDLSCs, respectively. 

For the immune suppression assay, human peripheral blood mononuclear cells (hPBMCs) were isolated from whole blood by density-gradient centrifugation over Ficoll-Hypaque (Sigma-Aldrich, St. Louis, MO, USA), with written informed consent under an Institutional Review Board (IRB)-approved protocol (Inha University Hospital IRB #11–104). hPBMCs were co-cultured with hPDLSCs (cell number ratio of hPDLSCs:PBMCs = 1:5) for three days with 1 μg/mL phytohemagglutinin (PHA; Sigma-Aldrich) [43] per 2×105 hPBMCs. After addition of [3H]-thymidine (1 μCi/well [0.037 MBq]) and incubation for an additional 16 h, T cell proliferation was determined with a 1450 MicroBeta TriLux (Perkin Elmer, Boston, MA, USA) based on incorporated radioactivity in counts per minute (CPM).

### 4.3. rhBMP-2 Pre-Treatment and Osteogenic Differentiation

After the development of hPDLSC sheets on thermoresponsive culture dishes, hPDLSC sheets were treated with 100 ng/mL rhBMP-2 (CGBio, Seoul, Republic of Korea) in osteogenic differentiation medium (ODM) for 4, 7, 14, or 21 days (Figure 1A) [42]. At each period of osteogenic differentiation, specific protein expression from the hPDLSC sheets were analyzed by Western blot with antibodies against RUNX2, alkaline phosphatase (ALP), osteocalcin (OCN), and cementum protein-1 (CEMP-1). All antibodies were obtained from Santa Cruz Biotechnology, Dallas, TX, USA, and glyceraldehyde 3-phosphate dehydrogenase (GAPDH) was also obtained (Cell Signaling, Danvers, MA, USA). Mineralized nodule formation by differentiated hPDLSCs was quantified with 2% alizarin red S (Sigma-Aldrich) after four weeks. Then, the stained nodules were treated with 10% cetylpyridinium chloride (2 mL/well) overnight and the optical density (O.D.) was measured at 540 nm on a microplate reader (Molecular Devices, San Jose, CA, USA).

### 4.4. Micro/Macro-Porous Biphasic Calcium Phosphate (MBCP) Cylindrical Block

The cylindrical MBCP blocks were composed of hydroxyapatite (HA) and β-tricalcium phosphate (β-TCP) in a 2:8 ratio. The size of the MBCP blocks were 8 mm in diameter and 3 mm height, with 80% global porosity (MBCP plus, Biomatlante, Vigneux de Bretagne, France). The cylindrical design, composition, and porosity of the MBCP blocks were planned to mimic structure of tooth dentin, where the hPDLSC sheets were applied for the regeneration of cementum and ligaments [7,34,44].

### 4.5. Transplantation of rhBMP Pretreated hPDLSC Sheets with MBCP Blocks In Vivo

After assembly of three-layered hPDLSC sheets and MBCP blocks, two dorsal pocket sites per mouse were randomly assigned for subcutaneous transplantation (Figure 3A, the most right). Two pockets were created using nine immunocompromised mice (six-week-old male Balb/c nude mice, OrientBio, Sungnam, Korea) for three groups (sample *n*-value = 6/group): control (MBCP block only), group A (hPDLSC sheet-MBCP block), and group B (rhBMP-2-hPDLSC sheet-MBCP block with pretreated hPDLSC sheets with 100 ng/mL rhBMP-2 in ODM for 14 days). All animals were euthanized with CO2 prior to specimen harvesting at four weeks. The ethics of the procedures and the scientific care of the animals were approved by the Institutional Animal Care and Use Committee of Jeonbuk National University Hospital, Jeonju, Korea (Approval No. CUH-IACUC-151218-1). 

### 4.6. Cell Survival of the Transplanted hPDLSC Sheets In Vivo

The hPDLSC sheets were prepared with or without 100 ng/mL rhBMP2 in ODM for 14 days, and then they were transfected with the pGL4.51 luciferase expression vector (Promega, Madison, WI, USA). The luciferase-expressing hPDLSC sheets were employed for transplantation in a total of three mice as described above. A luciferin (VivoGlo Luciferin; Promega) solution was injected intraperitoneally, and images were captured at 0, 1, 4, 7, 14, 21, and 28 days with an In-Vivo FX PRO imaging system (Bruker, Billerica, MA, USA).

### 4.7. Histomorphometric and Immunohistochemical Examinations for Tissue Regeneration

After sample decalcification, paraffin-embedded specimens were sectioned at 4 μm for histology. H&E staining was performed for histomorphometric analysis of vascular, mineralized, and fibrous tissue formation. Image-Pro Plus (Media Cybernetics, Rockville, MD, USA) was used, and tissue formation was quantified in terms of the area (μm^2^) of newly formed tissue and mineralized tissue around the MBCP blocks. PicroSirius red staining was used to assess collagen fiber formation around newly formed mineralized layers and deposited collagen. For the semi-quantification of collagen formation in three groups, a gradient visualization of collagen positivity showed density profiles in a colorimeter (CaseViewerTM, 3Dhistech, Budapest, Hungary). For immunohistochemistry, antibodies against human mitochondrial ribosomal protein L11 (hMito; Abcam, Cambridge, UK), ALP, OCN, or CEMP-1 (Santa Cruz Biotechnology) were stained as in a previously reported protocol [6]. Stained sections were analyzed with a confocal laser scanning microscope (Leica Microsystems, Wetzlar, Germany). 

### 4.8. Statistical Analysis

The SPSS ver. 21.0 software package was used for statistical analysis (IBM Corporation, Armonk, NY, USA). The Shapiro–Wilk test was performed to determine whether the data were normally distributed. Analysis of variance (ANOVA) or Student’s *t* tests were used for investigation of significant differences among or between the groups. The Kruskal–Wallis test or Mann–Whitney U test were used when the samples did not follow normal distribution. For post-hoc multiple comparisons, Tukey’s test for ANOVA or Dunn’s test for the Kruskal–Wallis test was used. The level of statistical significance was set at *p* < 0.05. Data are shown as mean ± standard deviation (SD). 

## Abbreviations

rhBMP-2	Human recombinant bone morphogenic protein-2
PDLSC	Periodontal ligament stem cells
NGM	Normal growth media
ODM	Osteogenic differentiation media
MBCP	Micro/macro-porous biphasic calcium phosphate
ALP	Alkaline phosphatase
OCN	Osteocalcin
CEMP-1	Cementum protein-1
RUNX2	Runt-related transcription factor 2
